# Impact of COVID-19 on Influenza and Pneumococcal Vaccination of Psoriatic Patients in Germany: Results from Vac-Pso

**DOI:** 10.3390/vaccines12060614

**Published:** 2024-06-04

**Authors:** Christian Kromer, Phoebe Wellmann, Daniel Kromer, Selina Patt, Johannes Mohr, Dagmar Wilsmann-Theis, Rotraut Mössner

**Affiliations:** 1 Department of Dermatology, University Medical Center Göttingen, 37075 Göttingen, Germany; christian.kromer@med.uni-goettingen.de (C.K.); phoebe.wellmann@med.uni-goettingen.de (P.W.); johannes.mohr@med.uni-goettingen.de (J.M.); 2 Real-World and Advanced Analytics, Ingress-Health HWM GmbH—A Cytel Company, 10963 Berlin, Germany; daniel.kromer.econ@gmail.com; 3 Department of Dermatology and Allergy, University Bonn, 53127 Bonn, Germany; selina.klein@ukbonn.de (S.P.); dagmar.wilsmann-theis@ukbonn.de (D.W.-T.)

**Keywords:** vaccination, COVID-19, immune system, epidemiology

## Abstract

Background: Suboptimal influenza and pneumococcal vaccination rates have been reported before the COVID-19 pandemics in certain populations at risk for severe infection. The aim of this longitudinal cohort study was to investigate changes in influenza and pneumococcal vaccination rates and patient perceptions in patients with psoriasis (PsO) before and during the pandemic. Methods: Data on vaccination, patient and disease characteristics, comorbidity, and patient perceptions were collected with questionnaires before and during the pandemic approximately one year later. Results: Over the whole cohort who participated in the follow-up visit (n = 287; 59.2% male; mean age: 56.3 years), both influenza and pneumococcal lifetime vaccination prevalences increased significantly from 50.5% to 66.2% and from 16.0% to 41.5%, respectively. A total of 88.5% of PsO patients were interested in a COVID-19 vaccination or had already received it. The reasons for and against vaccinations changed significantly before and during the pandemic. Conclusions: Despite a promising increase in the vaccination prevalence in our PsO cohort, it remains important that awareness for vaccinations is encouraged and closely monitored in future research, particularly in populations at risk.

## 1. Introduction

Psoriasis (PsO) is a common chronic inflammatory skin disorder, affecting approximately 2–4% of the population in European countries [1]. Several important medical conditions are associated with PsO, such as cardiovascular diseases, chronic kidney diseases, psoriatic arthritis (PsA), obesity, and metabolic disorders [2,3]. The therapeutic armamentarium constitutes of a wide range of topical, photo(chemo)-, and systemic therapies. Current guidelines recommend systemic immunosuppressive and immunomodulatory agents, such as conventional immunosuppressive agents, new small molecules, and biologics in patients with moderate to severe disease manifestations [4]. The presence of comorbid diseases and the application of immunosuppressive and/or immunomodulatory treatments in a considerable amount of PsO patients constitutes a risk for contracting infectious diseases, such as pneumonia, as well as for a more complicated course of the infectious disease [5,6,7,8]. Respiratory infections may be caused by a large variety of microorganisms, such as the bacterium *Streptococcus pneumoniae* and the respiratory viruses influenza and more recently COVID-19 [9,10]. Vaccinations are effective in reducing the risk as well as the severity of respiratory infections caused by pneumococci, influenza, and COVID-19 [11,12,13]. The German Standing Committee on Vaccination (STIKO) recommends pneumococcal, influenza, and COVID-19 vaccination, among others, for individuals over 60 years of age, patients with congenital or acquired immunodeficiency and those with underlying chronic diseases such as chronic heart or lung diseases, metabolic diseases, and chronic kidney or liver diseases [14]. Therefore, though not explicitly mentioned as an indication by the STIKO, a substantial number of PsO patients, including those under 60 years of age, meet the STIKO’s criteria for vaccination indication due to comorbid diseases and/or immunosuppressive therapy.

Before the onset of the COVID-19 pandemic, we investigated the influenza and pneumococcal vaccination rates of PsO patients and found suboptimal vaccination uptake for pneumococci and influenza [15,16]. As the COVID-19 pandemic has heightened the public awareness of respiratory infections and their respective vaccinations, the present follow-up study aimed to investigate changes in influenza and pneumococcal vaccination rates during the COVID-19 pandemic in our cohort of PsO patients. Moreover, an in-depth analysis of patient perceptions and barriers to vaccination for preventable respiratory infections was performed.

## 2. Materials and Methods

### 2.1. Study Design

In this longitudinal cohort study, PsO patients from the outpatient and inpatient clinics of the University Medical Center Göttingen and University Medical Center Bonn were enrolled. An initial study was conducted between August 2019 and March 2020, that is, before the COVID-19 pandemic. To evaluate the potential influences of the COVID-19 pandemic on influenza and pneumococcal vaccination prevalence in PsO patients, the study period was extended by an additional visit about one year after the initial visit. All patients who presented again between March and August 2021 at the two clinics and gave written consent were included in the follow-up.

The inclusion criteria have been described previously [15,16]. Briefly, they included adult patients with a dermatologist-diagnosed PsO. The study was performed according to the principles of the Declaration of Helsinki and approved by the Ethics Committees of all participating sites (Göttingen 40/2/19).

### 2.2. Data Collection

Data collection for the first visit was performed between August 2019 and March 2020. The collection of follow-up data was carried out from March 2021 until August 2021. Initial data were collected using a paper-based survey and an analysis of vaccination certificates, if available. In the follow-up, data were retrieved via a paper-based questionnaire. The re-evaluation of vaccination records at this subsequent point was not performed, as the patients’ ability to reliably report vaccinations performed during the follow-up period was assumed.

In the questionnaire handed out at baseline and follow-up, patients were asked to give information on sociodemographic characteristics such as age, sex, and partnership status, occupational status, disease characteristics (disease duration, family history of PsO, disease severity (Psoriasis Area and Severity Index (PASI)), health-related quality of life (Dermatology Life Quality Index (DLQI)), current and former dermatological therapy, and comorbidity (PsA, atopic, metabolic, psychiatric, and neoplastic diseases, and smoking status). Additionally, patients’ history of infectious diseases (pneumonia, bronchitis, and herpes zoster) was recorded. Medical records were consulted to supplement the provided information on comorbidity, course of therapy, and disease characteristics.

In both surveys, the individual vaccination status for the pneumococcal and influenza vaccination was gathered. As influenza vaccination is a yearly recommended vaccination and may not be sufficiently documented in vaccination records, both patients with documentation of the vaccination and those with self-reported vaccinations were considered as vaccinated. For pneumococcal vaccination, only those with the vaccination documented in their vaccination records were assumed to be vaccinated in the initial study, whereas in the follow-up a self-reported positive vaccination status was accepted. Additionally, patients were asked to provide information on their COVID-19 vaccination status in the follow-up. Moreover, study participants disclosed information in the questionnaire concerning the factors influencing their decision whether to receive the before-mentioned vaccinations. This information was derived from the individuals who indicated having received vaccinations in the questionnaire. As COVID vaccinations had not been accessible for all patients at the time of the follow-up visit, their intention to receive such a vaccination was also asked for.

### 2.3. Statistical Analysis

The statistical analysis was conducted with SPSS Statistics (version 28.0.1, IBM, Armonk, NY, USA). The cohort characteristics, as well as data on vaccination prevalence and patient perception, were analyzed descriptively. The normally distributed continuous variables were summarized as mean with standard deviation (SD), and the not normally distributed variables as median with interquartile range (IQR). Fisher’s exact test was used to identify statistically significant association between categorical variables. Statistical significance was assumed at *p*-values < 0.05.

## 3. Results

### 3.1. Cohort Characteristics

Initially, 322 PsO patients from Bonn and Göttingen participated in the baseline period of data collection, and 287 of those could be recruited for the follow-up study, resulting in a participation rate of 89.1% in the follow-up (Table 1). The following analysis was based on those who participated in both visits. More than half of the patients were male (59.2%). The average age was 56.3 years. The follow-up visit was performed on average 1.6 years after the initial visit. Both at baseline and follow-up assessments, more than half of the patients were employed (56.8% and 59.2%, respectively). Retirees constituted the second largest occupational group, representing approximately one-third of patients (33.4% and 35.2% at baseline and follow-up, respectively). Among the employed, the majority of patients worked either full-time or part-time, with 71.8% and 22.1% at baseline, and 61.8% and 24.7% at follow-up, respectively.

Concomitant PsA was present in more than half of all patients, both at baseline (60.6%) and follow-up (62.4%) (Table 2). There was an increase in cardiovascular diseases, with 17.8% affected at baseline and 20.9% at follow-up. Similarly, the prevalence of diabetes mellitus was found to be lower at baseline with 12.9%, compared to 15.0% at follow-up. Chronic kidney disease was present in 2.4% of patients at baseline and 4.2% at follow-up. A lower percentage of patients reported suffering from depression at baseline compared to follow-up (20.6% vs. 22.6%). Smoking behavior varied little between baseline and follow-up. Furthermore, an overall rise in the lifetime prevalence of infectious diseases between baseline and follow-up was reported, with the lifetime prevalence of pneumonia increasing from 17.4% to 18.1%, and of bronchitis from 30.0% to 32.4%, as well as of herpes zoster from 18.1% to 19.5%, respectively.

The mean PASI score for the follow-up cohort decreased from baseline at 3.4 to 2.3 at follow-up, and the mean DLQI from 5.3 to 4.0.

The total number of patients with a current systemic therapy for PsO decreased from 94.4% at baseline to 91.6% at follow-up, and 15.0% had switched their current systemic therapy between baseline and follow-up. With respect to baseline, patients were less frequently treated with TNF-alpha antagonists at follow-up (21.3% and 18.5%, respectively) and IL-17-(R)-antagonists (19.5% and 18.5%, respectively), while the proportion of patients treated with IL-(12)/23 antagonists increased from 36.2% to 40.8%.

The follow-up data analysis showed that after the onset of the COVID-19 pandemic, a total of eleven patients (3.8%) tested positive for COVID-19 infection. Among those, seven patients (63.6%) reported infection-related symptoms. Out of the symptomatic cases, six patients (85.7%) received outpatient treatment, while one patient (14.3%) received intensive care unit (ICU) treatment for COVID-19 infection.

### 3.2. Influenza Vaccination

At baseline, 50.5% of PsO patients had received at least one influenza vaccination in their lifetime and 35.5% of all patients had received vaccinations in the current or the past year (covering one to two vaccination seasons, depending on the visit date, Table 3). During the interval between baseline and follow-up visit, 56.1% underwent vaccination in at least one of the corresponding vaccination seasons (2019/2020 and 2020/2021, depending on date of data collection), which was significantly more than at baseline (*p* < 0.001). Among those, 28.0% received their first influenza vaccination ever, and 14.3% of patients received both influenza and pneumococcal vaccinations for the first time in their lifetime.

Influenza immunization coverage was lowest in the younger PsO patients (Table 3). Overall, the proportion of patients with influenza vaccinations at follow-up examination was consistently higher in all age groups compared to baseline. The vaccination rate increased to 79.5% in the age group of 70–79 years and to 100.0% in the age group of >80 years. The increase between baseline and follow-up was significant in the age groups 50–59 years (27.8% to 52.1%; *p* < 0.001) and 60–69 years (52.9% to 70.4%; *p* = 0.024).

Vaccination was most frequently recommended by the general practitioner (73.0% and 67.7% at baseline and follow-up, respectively). At follow-up, a significantly higher proportion of patients received a recommendation for a vaccination by a dermatologist compared to baseline (34.2% vs. 12.0%; *p* < 0.001).

### 3.3. Pneumococcal Vaccination

According to the vaccination certificates, the number of patients vaccinated against pneumococci at baseline amounted to 46 individuals (16.0%; Table 4). At follow-up, 99 patients (34.5%) reported to have received vaccination against pneumococci after the baseline visit, and in 73 patients (61.3%) it was the first pneumococcal vaccination ever. Thus, the lifetime prevalence significantly increased to 119 patients (41.5%; *p* < 0.001).

Stratification according to age groups showed a higher prevalence with higher age at baseline. At follow-up, a significant increase in vaccinations compared to baseline was found in the age groups 40–49 years (24.3% vs. 4.7%; *p* = 0.002), 50–59 years (39.6% vs. 13.4%; *p* < 0.001), 60–69 years (54.9% vs. 26.5%; *p* = 0.001), and 70–79 years (61.5% vs. 30.0%; *p* = 0.015).

General practitioners recommended the performed pneumococcal vaccination for 71.7% of patients at baseline and 61.6% at follow-up (*p* = 0.266), while dermatologists suggested vaccinations to 13.9% of patients at baseline and 50.5% at follow-up (*p* < 0.001).

### 3.4. COVID-19 Vaccination

A majority of PsO patients either expressed their intent to receive a COVID-19 vaccination or had already been vaccinated against COVID-19 (88.5%; Table 5). Among the patients who were positively inclined towards a COVID-19 vaccination, the majority had also received immunization against influenza (20.9%), pneumococci (4.3%), or both (41.3%). The majority of patients refusing COVID-19 vaccination were unvaccinated against both pneumococci and influenza (28/33; 84.8%).

### 3.5. Reasons for and against Vaccination

Among the patients vaccinated against influenza, 39 (39.0%) stated at baseline that the physician’s advice had an impact on their decision for the vaccination (Table 6). At follow-up, this number showed a significant increase to 106 (65.8%) (*p* < 0.001). The importance of the treatment of the patient’s skin disease in the decision-making also grew significantly from 12 patients (12.0%) at baseline to 44 (27.3%) at follow-up (*p* = 0.003). In addition, 40 (24.8%) participants at follow-up mentioned the COVID-19 pandemic as an important reason for the influenza vaccination.

The absence of a vaccination recommendation by a physician was a major factor for patients’ decision not to receive influenza vaccination at baseline (n = 63; 33.7%) and follow-up (n = 41; 32.5%). A total of 93 patients (49.7%) at baseline deemed the vaccination not necessary, whereas this reason was significantly less frequently mentioned at follow-up (n = 41, 32.5%; *p* = 0.004). A lacking history of severe flu was another frequently mentioned reason against vaccination at baseline (n = 71; 38.0%) and even proportionally more at follow-up (n = 63; 50.0%; *p* = 0.037).

The reasons for and against pneumococcal vaccination and their alteration during the pandemic were comparable (Supplementary Table S1).

## 4. Discussion

This study aimed to identify the influence of the COVID-19 pandemic on influenza and pneumococcal vaccination prevalence in PsO patients. Overall, a significant increase in prevalence for both vaccinations could be observed, with a pneumococcal lifetime vaccination prevalence rising from 16.0% to 41.5% and influenza lifetime prevalence increasing from 50.5% to 66.2%.

To our knowledge, there are currently no studies investigating the pre and post COVID-19 influenza or pneumococcal vaccination rates in PsO patients. However, in other populations at risk, the influence of the COVID-19 pandemic on vaccination behavior has been investigated. Fragoulis and colleagues compared the influenza vaccination rate in autoimmune rheumatic disease patients in the pre-COVID vaccination season of 2019/2020 to the season after the onset of the COVID-19 pandemic in the season 2020/2021 [17]. The authors observed an increase in vaccination coverage (prevalence: 76% to 83%). The higher initial vaccination rate may be partly explained by the different design, cultural differences, and possibly higher awareness of the immune-mediated disease as a risk factor. Scardina and colleagues focused on the changes in the influenza vaccination coverage of Italian healthcare workers between the vaccination seasons 2018/2019, 2019/2020 and 2020/2021 [18]. Influenza vaccination prevalence in the two vaccination seasons before the COVID-19 pandemic remained low at 10.2% in 2018/2019 and 11.9% in 2019/2020, respectively, but nearly quadrupled to 39.3% in 2020/2021.

There are only few data on pneumococcal vaccination coverage during the COVID-19 pandemic. Janssens and colleagues reported an increase in pneumococcal vaccination in Danish at-risk patients from 18.2% in 2018 to 23.6% in 2021 [19]. A Turkish study from 2021 analyzed vaccination rates and attitudes towards the vaccination of cancer patients in a tertiary treatment center before and after the onset of the COVID-19 pandemic [20]. Pneumococcal vaccination prevalence slightly increased from 15.8% before the pandemic to 18.1% during the pandemic, with 43.0% of patients stating that they were considering getting a pneumococcal vaccination due to the COVID-19 pandemic. Similarly, the COVID-19 pandemic was identified as a key reason for influenza and pneumococcal vaccinations in our cohort. Conclusively, we found that patients who were willing to get vaccinated against COVID-19 were also more likely to be vaccinated against influenza and pneumococci. The impact of the pandemic on individual perceptions increasing vaccination awareness and uptake has recently been reported by others [21,22,23,24].

In our cohort, influenza and pneumococcal vaccination were most commonly recommended by the patient’s general practitioner at baseline and at follow-up. Conversely, the lack of a doctor’s recommendation was reported as the most common reason for the decision against the vaccination. Similar trends concerning the reasons for and against pneumococcal and influenza vaccination have been reported in a study among elderly people from Singapore [25]. A total of 83.4% of vaccinated individuals followed the advice of healthcare professionals to receive the influenza vaccination, while 48.1% of those not vaccinated stated the lack of information as an influencing factor in their decision-making. This is consistent with the findings of Ergin and colleagues, where patients mentioned not having enough knowledge about the recommended vaccines (44.0%) and not receiving a recommendation from their doctor (61.5%) as the main contributors to their decision not to get vaccinated [21]. Zens et al. reported that 70.1% of at-risk patients listed “not enough information about the topic” as a reason against a pneumococcal vaccination [26]. A Greek study from 2021 also described the main reason against pneumococcal vaccination being the lack of recommendation by the caring physician (82.5%) whereas a multi-variate analysis showed a significant correlation between at-risk patients being well informed about the vaccine and a positive pneumococcal vaccination status [27]. In summary, our data, in line with the literature, underlines the general practitioners’ pivotal role in vaccination counseling and management.

Interestingly, significantly more patients indicated the dermatologist as the source of recommendation for vaccination. This gives hope to an increased awareness for vaccination of patients at risk among dermatologists.

A major strength of our study is its longitudinal design, with an analysis of vaccination and patients’ perceptions before and during the COVID-19 pandemic and a high participation rate in the follow-up, minimizing selection bias.

The following limitations should be taken into consideration. First, the patient cohort was limited to those with more severe PsO and more often accompanying PsA, as they were recruited in specialized tertiary care centers. The distribution of the patient collective between the two study centers was skewed, with the majority of patients recruited in one of the centers. The patients were required to self-report some information, which can lead to recall bias and risk of imprecise responses. The study observes an increase in vaccination awareness during the study period, However, it was not examined whether there was a general increase in vaccination awareness prior to the COVID-19 pandemic. Moreover, vaccination intentions and real-life vaccinations of COVID-19 vaccination might differ, and we did not collect data on actual COVID-19 vaccination at a later point of time. Another limitation of the study is that patients in this follow-up period were only asked about the presence of a pneumococcal vaccination, while further details regarding the specific type of vaccine received were only collected at the baseline visit, as described in [16].

## 5. Conclusions

In conclusion, our data, along with findings from other research papers, consistently demonstrate an increase in vaccination prevalence both during and after the COVID-19 pandemic, with pneumococcal vaccination prevalence in our patient cohort rising from 16.0% to 41.5% and influenza vaccination prevalence from 35.5% to 56.1%. Several factors appear to contribute to this positive trend. Firstly, the pandemic itself raised awareness about the importance of vaccination. Media coverage and public discourse potentially played an important role in emphasizing the value of vaccinations. Additionally, the pandemic drew attention to broader health and infection prevention issues, further highlighting the need for widespread immunization against pneumococci and influenza to protect vulnerable, at-risk populations. Although COVID-19 led to an increase in vaccination prevalence, it becomes apparent that an increased effort is required to sustain and further enhance these rates. Therefore, primary care physicians play a crucial role in educating patients and administering vaccinations, given the significant influence of physician advice on patients’ vaccination decisions. Future initiatives should focus on continued education, comprehensive vaccination campaigns, and accessible vaccination options. This approach could represent a valuable opportunity to ensure that vaccination rates remain on the rise and contribute to better public health outcomes.

## Figures and Tables

**Table 1:** Cohort characteristics.

Characteristic	Baseline Visit ^a^, n (%)	Follow-Up Visit, n (%)
Female sex	117 (40.8)	117 (40.8)
Age, years, mean (SD)	54.7 (13.4)	56.3 (13.2)
**Occupational status**		
Working	163 (56.8)	170 (59.2)
Working full-time	117 (71.8)	105 (61.8)
Working part-time	36 (22.1)	42 (24.7)
Currently in home office	n/a	10 (5.9)
Self employed	10 (6.1)	13 (7.6)
**Working in critical infrastructure ^b^**	n/a	103 (60.6)
Medical/Geriatric care	n/a	40 (38.8)
Infrastructure	n/a	20 (19.4)
Craftmanship	n/a	12 (11.7)
Education	n/a	7 (6.8)
Public service	n/a	7 (6.8)
Food industry	n/a	6 (5.8)
Other services	n/a	11 (10.7)
Currently unemployed	8 (2.8)	7 (2.4)
Student	11 (3.8)	4 (1.4)
Retired	96 (33.4)	101 (35.2)
Unable to work	9 (3.1)	5 (1.7)
**Partnership status**		
In a permanent relationship	220 (76.7)	218 (76.0)
Single	52 (18.1)	51 (17.8)
Widowed	15 (5.2)	18 (6.3)

^a^ Only patients who also participated in the follow-up visit were considered (n = 287). ^b^ Data were only collected in the follow-up visit. Patients indicated themselves whether they numbered their profession among critical infrastructure. n/a: Data were only collected in the follow-up visit.

**Table 2:** Comorbidity.

Comorbidity	Baseline Visit, n (%)	Follow-Up Visit, n (%)
PsA	174 (60.6)	179 (62.4)
Age at onset of PsA, years, mean (SD)	46.7 (13.3)	47.5 (12.3)
Arterial hypertension	131 (45.6)	141 (49.1)
Cardiovascular disease	51 (17.8)	60 (20.9)
Dyslipidemia	78 (27.2)	86 (30.0)
Hepatic steatosis	94 (32.8)	99 (34.5)
Hepatic cirrhosis	7 (2.4)	8 (2.8)
Other hepatic diseases	13 (4.5) ^a^	14 (4.9) ^b^
Diabetes mellitus	37 (12.9)	43 (15.0)
Thereof insulin-dependent	9 (24.3)	10 (23.3)
Chronic kidney disease	7 (2.4)	12 (4.2)
COPD/emphysema	30 (10.5)	32 (11.1)
Asthma	27 (9.4)	30 (10.5)
Allergic contact dermatitis	24 (8.4)	28 (9.8)
Allergic rhinoconjunctivitis	47 (16.4)	50 (17.4)
Food allergies	24 (8.4)	27 (9.4)
Depression	59 (20.6)	65 (22.6)
Neoplastic diseases	10 (3.5) ^c^	13 (4.5) ^d^
**Smoking status**		
Current smoker	96 (33.4)	95 (33.1)
Ex-smoker	128 (44.6)	129 (44.9)
Never smoker	63 (22.0)	63 (22.0)
**Previous infectious diseases**		
Pneumonia	50 (17.4)	52 (18.1)
Hospitalization due to pneumonia	14 (28.0)	16 (30.8)
Bronchitis	86 (30.0)	93 (32.4)
Hospitalization due to bronchitis	7 (8.1)	9 (9.7)
Herpes zoster	52 (18.1)	56 (19.5)
(Post)herpetic neuralgia	40 (76.9)	45 (80.4)
**Disease activity**		
PASI mean (SD) ^e^	3.4 (5.7)	2.3 (3.1)
PASI median (IQR) ^e^	1.5 (0.0–3.0)	1.6 (0.0–4.0)
DLQI mean (SD)	5.3 (6.5)	4.0 (5.3)
DLQI median (IQR)	2.0 (0.0–6.0)	3.0 (0.0–8.0)

^a^ Focal nodular hyperplasia (n = 1), hemangioma (n = 2), hepatitis C (n = 1), Gilbert’s syndrome (n = 1), primary biliary cholangitis (n = 1), healed hepatitis B (n = 4), healed hepatitis C (n = 1), healed hepatitis E (n = 2). ^b^ Focal nodular hyperplasia (n = 1), hemangioma (n = 2), hepatitis C (n = 1), hepatitis E (n = 1), Gilbert’s syndrome (n = 1), primary biliary cholangitis (n = 1), healed hepatitis B (n = 4), healed hepatitis C (n = 1), healed hepatitis E (n = 2). ^c^ B-cell-lymphoma (n = 2), cervix carcinoma (n = 2), malignant melanoma (n = 2), breast cancer (n = 3) and renal cell carcinoma (n = 1). ^d^ B-cell-lymphoma (n = 2), cervix carcinoma (n = 2), malignant melanoma (n = 3), breast cancer (n = 3), gastric cancer (n = 1), bronchial carcinoma (n = 1) and renal cell carcinoma (n = 1). ^e^ Data on PASI were missing in 41 patients.

**Table 3:** Influenza vaccination.

Characteristic	Baseline Visit, n (%)	Follow-Up Visit, n (%)	*p*-Value
**Lifetime prevalence**	**145 (50.5)**	**190 (66.2)**	**<0.001**
Vaccination during the last two seasons ^a^	**102 (35.5)**	**161 (56.1)**	**<0.001**
First vaccination ever during the last two seasons	n/a	45 (28.0)	
**Vaccination rate according to age groups (years of age)** ^a^			
18–29	1 (6.7)	3 (23.1)	0.311
30–39	3 (10.3)	6 (23.1)	0.281
40–49	14 (32.6)	16 (43.2)	0.361
50–59	**27 (27.8)**	**50 (52.1)**	**<0.001**
60–69	**36 (52.9)**	**50 (70.4)**	**0.024**
70–79	18 (60.0)	31 (79.5)	0.109
≥80	3 (60.0)	5 (100.0)	0.444
**Recommendation for vaccination** ^a,b^			
General practitioner	73 (73.0)	109 (67.7)	0.407
Dermatologist	**12 (12.0)**	**55 (34.2)**	**<0.001**
Rheumatologist	12 (12.0)	19 (11.8)	1.000
Other physician	8 (8.0)	12 (7.5)	1.000
Patient has informed themself	23 (23.0)	35 (21.7)	0.879
Friends/Family	1 (1.0)	3 (1.9)	1.000

^a^ Only influenza vaccinations from the year of the survey and the previous year (approximately the last two vaccination seasons) were considered at baseline due to influenza being a seasonal vaccination. At follow-up, patients who reported a vaccination after the baseline visit in the two vaccination seasons 19/20 and/or 20/21 were considered vaccinated. Subgroup analysis is based on these data. ^b^ Data were based on patients who had indicated vaccination in the questionnaire (n = 100 at baseline and n = 161 at follow-up). Multiple answers were permitted. Significant differences are printed in bold. n/a: not applicable.

**Table 4:** Pneumococcal vaccination.

Characteristic	Baseline Visit, n (%)	Follow-Up Visit, n (%)	*p*-Value
Lifetime prevalence	**46 (16.0)**	**119 (41.5)**	**<0.001**
First vaccination ever ^a^	n/a	73 (61.3)	
**Vaccination rate according to age groups (years of age)**			
18–29	0 (0.0)	0 (0.0)	1.000
30–39	2 (6.9)	5 (19.2)	0.236
40–49	**2 (4.7)**	**9 (24.3)**	**0.020**
50–59	**13 (13.4)**	**38 (39.6)**	**<0.001**
60–69	**18 (26.5)**	**39 (54.9)**	**0.001**
70–79	**9 (30.0)**	**24 (61.5)**	**0.015**
≥80	2 (40.0)	4 (80.0)	0.524
**Recommendation for vaccination** ^b^			
General practitioner	33 (71.7)	61 (61.6)	0.266
Dermatologist	**5 (13.9)**	**50 (50.5)**	**<0.001**
Rheumatologist	4 (11.1)	6 (6.1)	0.456
Other physician	2 (5.6)	7 (7.1)	1.000
Patient has informed themself	2 (5.6)	4 (4.0)	0.657
Friends/Family	0 (0.0)	0 (0.0)	1.000

^a^ Patients with at least one pneumococcal vaccination in their lifetime were considered vaccinated. Pneumococcal vaccination between baseline and follow-up visit comprised at least one vaccination in either or both of the vaccination seasons 2019/2020 and 2020/2021. ^b^ Data were based on patients who had indicated vaccination in the questionnaire (n = 36 at baseline and n = 99 at follow-up). Multiple answers were permitted. Significant differences are printed in bold. n/a: not applicable.

**Table 5:** COVID-19 vaccination.

Characteristic	n (%)
**COVID-19 vaccination performed or planned**	**254 (88.5)**
Thereof neither vaccinated against pneumococci nor influenza	85 (33.5)
Thereof only vaccinated against influenza	53 (20.9)
Thereof only vaccinated against pneumococci	11 (4.3)
**Thereof vaccinated against both pneumococci and influenza**	**105 (41.3)**
**COVID-19 vaccination not planned**	**33 (11.5)**
**Thereof neither vaccinated against pneumococci nor influenza**	**28 (84.8)**
Thereof only vaccinated against influenza	2 (6.1)
Thereof only vaccinated against pneumococci	2 (6.1)
Thereof vaccinated against both pneumococci and influenza	1 (3.0)

The largest subgroup in both groups is highlighted in bold. As not all patients had been offered a vaccination prior to the study date, we aggregated those who were vaccinated (72; 25.1%) and those who planned to get vaccinated (182; 63.4%) into one group.

**Table 6:** Reasons for and against influenza vaccination.

Reasons	Baseline Visit, n (%)	Follow-Up Visit, n (%)	*p*-Value
**Reasons for vaccination** ^a^			
Physician’s advice	**39 (39.0)**	**106 (65.8)**	**<0.001**
General recommendation	50 (50.0)	98 (60.9)	0.096
Comorbidity/comedication	16 (16.0)	27 (16.8)	1.000
Skin disease	8 (8.0)	25 (15.5)	0.086
Treatment of skin disease	**12 (12.0)**	**44 (27.3)**	**0.003**
Risk due to profession	n/a	23 (14.3)	
COVID-19 pandemic	n/a	40 (24.8)	
Due to this survey bringing the vaccination to the patient’s attention	n/a	9 (5.6)	
Other reasons	**15 (15.0)** ^b^	**5 (3.1)** ^c^	**0.001**
**Reasons against vaccination** ^d^			
Lacking recommendation by a physician	63 (33.7)	41 (32.5)	0.903
No personal history of severe flu	**71 (38.0)**	**63 (50.0)**	**0.037**
Vaccination deemed not necessary by patient	**93 (49.7)**	**41 (32.5)**	**0.004**
Lacking confidence in protective effect	42 (22.5)	23 (18.3)	0.397
Potential side effects	52 (27.8)	33 (26.2)	0.796
Patient forgot to get vaccinated	28 (15.0)	14 (11.1)	0.399
No time for vaccination	22 (11.8)	8 (6.3)	0.121
Advised not to get vaccinated by a physician	8 (4.3)	3 (2.4)	0.535
(Co)payment	4 (2.1)	n/a	
Inflammatory activity of skin disease	4 (2.1)	3 (2.4)	1.000
Treatment of skin disease	12 (6.4)	12 (9.5)	0.387
Comorbidity/comedication	13 (7.0)	9 (7.1)	1.000
COVID pandemic	n/a	9 (7.1)	
Other reasons	**1 (0.5)** ^e^	**8 (6.3)** ^f^	**0.004**

^a^ Data were based on patients who had indicated vaccination in the questionnaire (n = 100 at baseline and n = 161 at follow-up). Multiple answers were permitted. ^b^ Other reasons comprised “required by employer” (n = 9), “offered by employer” (n = 3), “frequent contact with people” (n = 3), and “experience with influenza infection in other people or themselves” (n = 4). ^c^ Other reasons comprised “experience with influenza infection in other people or themselves” (n = 3), “to protect others” (n = 1) and “current primary residence in South America” (n = 1). ^d^ Data were based on patients who had not indicated vaccination in the questionnaire (n = 187 at baseline and n = 126 at follow-up). Multiple answers were permitted. ^e^ Other reasons comprised “vaccine not available” (n = 1). ^f^ Other reasons comprised “vaccine not available” (n = 4), “had pneumonia as a child” (n = 1), “vaccination is planned” (n = 1), “patient had been vaccinated in the past and did not know that it is an annual vaccination” (n = 1) and “patient prefers frequent sauna visits in order to prevent influenza infection” (n = 1). n/a: not applicable.

## Data Availability

The data presented in this study are available upon reasonable request if the provision of the requested data is in accordance with the European data protection laws.

## Supplementary Materials

The following supporting information can be downloaded at: https://www.mdpi.com/article/10.3390/vaccines12060614/s1, Table S1: Reasons for and against pneumococcal vaccination.
