# Supplementary material for: Impact of COVID-19 on Influenza and Pneumococcal Vaccination of Psoriatic Patients in Germany: Results from Vac-Pso
Source: Vaccines (Basel). 2024 Jun 4;12(6):614. doi: 10.3390/vaccines12060614 (PMC11209491; doi:10.3390/vaccines12060614)
Supplement: Supplementary file 1 [file vaccines-12-00614-s001.zip › vaccines-3020769-supplementary.pdf]

Table S1. Reasons for and against pneumococcal vaccination

Reasons	Baseline visit, n (%)	Follow-up visit, n (%)	p-value
**Reasons for the vaccination** [a]			
Physician's advice	23 (63.9)	82 (82.8)	0.033
General recommendation	12 (33.3)	30 (30.3)	0.834
Comorbidity / comedication	8 (22.2)	15 (15.2)	0.437
Skin disease	3 (8.3)	18 (18.2)	0.191
Treatment of skin disease	7 (19.4)	31 (31.3)	0.200
Job related risks	-	9 (9.1)	
COVID-pandemic	-	27 (27.3)	
Due to this survey bringing the vaccination to the patient's attention	-	17 (17.2)	
Other reasons	1 (2.8) [b]	2 (2.0) [c]	1.000
**Reasons against vaccination** [d]			
Patient has been vaccinated in the past	n/a	26 (13.8)	
Lacking recommendation by a physician	174 (69.3)	92 (48.9)	<0.001
No personal history of severe flu/pneumonia	37 (14.7)	58 (30.9)	0.0001
Vaccination not deemed necessary by patient	33 (13.1)	21 (11.2)	0.560
Lacking confidence in protective effect	10 (4.0)	4 (2.1)	0.411
Potential side effects	25 (10.0)	10 (5.3)	0.108
Patient forgot to get vaccinated	21 (8.4)	19 (10.1)	0.616
No time for vaccination	7 (2.8)	6 (3.2)	1.000
Advised not to get vaccinated by a physician	4 (1.6)	2 (1.1)	1.000
(Co)payment	3 (1.2)	3 (1.6)	0.705
Inflammatory activity of skin disease	3 (1.2)	4 (2.1)	0.468
Treatment of skin disease	8 (3.2)	3 (1.6)	0.366
Comorbidity / comedication	5 (2.0)	3 (1.6)	1.000
COVID-pandemic	n/a	11 (5.9)	
Other reasons	18 (7.2) [e]	24 (12.8) [f]	0.070

[a] Data was based on patients who had indicated vaccination in the questionnaire (n=36 at baseline and n=99 at follow-up). Multiple answers were permitted.

[b] Other reasons comprised "secondary residence in South America (n=1) and "patient frequently suffers from pneumonia" (n = 1)

[c] Other reason comprised "required by employer" (n = 1).

[d] Data was based on patients who had not indicated vaccination in the questionnaire (n=251 at baseline and n=188 at follow-up). Multiple answers were permitted.

[e] Other reasons comprised "vaccination is planned" (n = 5), "patient did not know about the vaccine" (n = 4), "vaccine not available" (n = 12), "patient wants to wait until after the COVID-pandemic" (n = 1), "vaccination is not recommended for my patient group" (n = 1) and "patient was told that the vaccination is only recommended in patients over 50 years of age" (n = 1).

[f] Other reasons comprised "patient does not want to take too many pharmaceuticals" (n = 1), "vaccination is planned" (n = 3) and "patient did not know about the vaccine" (n = 14).

n/a: not applicable
